# On the Differential Diagnosis of Popliteal Swellings

**Published:** 1895-12-21

**Authors:** Alexander Miles

**Affiliations:** Surgical Tutor, Royal Infirmary, Edinburgh.


### MEDICAL PROGRESS AND HOSPITAL CLINICS.

[*The Editor will be glad to receive offers of co-operation and contributions from members of the profession. All letters should be addressed to* The Editor, at the Office, 428, Strand, London, W.C.]

### ON THE DIFFERENTIAL DIAGNOSIS OF POPLITEAL SWELLINGS.

By Alexander Miles, M.D., F.R.C.S. Ed., Surgical Tutor, Royal Infirmary, Edinburgh.

The differential diagnosis of the various swellings which occur in the popliteal space is by no means always an easy matter. If proof of this statement were needed it would readily be found in the fact that no less distinguished a surgeon than Pirogoff has recorded an instance in which he incised what proved to be a popliteal aneurism, in the belief that it was an abscess. Similar errors have frequently been reported, and doubtless if all operators were equally frank many more would have to be confessed.

For purposes of clinical consideration swellings in this region may be divided into four groups: (1) Inflammatory swellings, (2) swellings of blood vessels, (3) bursal swellings, and (4) new growths.

**I.—Inflammatory Swellings.**—No more difficulty will be experienced in recognising (*a*) a superficial collection of pus in this region than in any other part of the body, and the usual evidences, local and general, of suppuration will suffice. It is another matter however, when pus forms (*b*) underneath the popliteal fascia. This firm fibrous membrane obscures typical appearances, and the close proximity of the popliteal artery lends to the swelling adventitious signs, which cause it to assume some of the characters of an aneurism. In fact, it is these transmitted signs which give rise to most of the difficulty in the differential diagnosis of the conditions under consideration. In the present case, however—deep-seated suppuration—the history of some originating cause such as a wound, bruise, or antecedent severe illness, followed by the occurrence of a chill or rigor, with high temperature and rapid pulse and a general feeling of illness; and in addition a dull throbbing pain and tenderness on pressure, will indicate the nature of the swelling. The detection of fluctuation on relaxing the fascia, and the absence of expansion in the pulsatile tumour will in most cases remove all doubt. (*c*) Glandular inflammation or suppuration may give rise to difficulty. As the lymphatic glands which surround the popliteal artery receive afferent vessels almost entirely from the deep parts of the leg, infective inflammation of them is not common. It does, however, occur every now and then, as a few superficial afferent lymphatics accompany the short saphena vein through the popliteal fascia. The existence of painful and tender lumps deeply placed in the middle of the popliteal space, with or without a transmitted pulsation, but devoid of expansion, bruit or thrill, and following on some form of irritation, such as a septic wound, eczema, or ulcer in the area of distribution of the short saphena vein (the outer side of the foot, ankle, and leg), will characterise the condition. Should suppuration have ensued the clinical appearances will come to resemble those of deep-seated cellular inflammation last described, and the same diagnostic points will arise. (*d*) A cold abscess does rarely occur in the popliteal space. The presence of fluctuation, usually superficial, occurring in a tubercular subject, the bluish colour of the skin and the absence of the ordinary signs and symptoms of active suppuration in most cases indicates the diagnosis. Any pulsation will obviously be transmitted, and is unlikely to raise doubt.

It is not to be forgotten that pus may form in or around any of the swellings of the other groups—aneurysmal, bursæ, or tumours—when all the acute symptoms of suppuration will be superadded to the appearances proper to these respective conditions. The difficulties of diagnosis are then enormously increased, and nothing but the greatest care and patience in the investigation of the previous history of the patient and his condition will prevent serious and even fatal errors.

In connection with pulsation as a sign it is to be borne in mind that there are some rare cases even of aneurism in which it is not to be detected. Pirogoff's case, already referred to, was a case in point. Its absence, therefore, although presumptive evidence of the inflammatory nature of a given swelling, is not conclusive.

**II.—Swellings of Blood Vessels.**—Undoubtedly the most important condition to exclude with certainty in forming a diagnosis of a popliteal swelling is an aneurism of the popliteal artery. This is usually difficult, because the subjacent artery gives to most of the other affections appearances which cause them closely to resemble an arterial dilatation. The cardinal sign of an aneurism is that the tumour not only has an up and down pulsation, but that it is at the same time *expansile*. Its dilating in all directions may best be demonstrated clinically by placing the two hands flat on the swelling, and observing that the hands are not only raised with each beat of the pulse, but that the interval between them is appreciably increased during the systole. A very slight degree of expansion can readily be appreciated by this method. Again, pressure over the artery, proximal to the swelling, in addition to arresting the pulsation, which, of course, it does in all pulsatile swellings, causes a marked diminution in the size of the tumour, while the relaxation of pressure is immediately followed by its reappearance. The pulse beyond the aneurism, *e g.*, in the anterior or posterior tibial artery, on the affected side, is smaller and somewhat delayed by comparison with that of the healthy side. A very distinct bruit is heard, and a thrill felt over the swelling in most cases. General œdema beyond, with dilatation of the veins, is not infrequent. Pain of a sharp lancinating or dull boring character is a prominent feature in the later stages of aneurism. This is due either to involvement of nerves or erosion of bone by the pressure of the swelling. Other points bearing on the diagnosis of aneurism are the age and occupation of the patient; the mode of onset of his trouble, whether inflammatory or not; and his previous history as to venereal disease, syphilis being one of the chief predisposing factors in the causation of aneurism. Other pulsatile swellings in this region will differ from aneurism in that they are not expansile; proximal pressure on the main artery does not affect their size; the pulse beyond is not altered; and there is, if any, only a very indistinct bruit. Another vascular swelling which sometimes occurs in the popliteal space is that caused by (*b*) a varicose condition of the external saphenous vein. Little difficulty will be experienced in diagnosing this condition if its superficial character, its area of distribution, and the effect of position and of distal pressure on the vein be observed.

**III. Bursal Swellings.**—The popliteal region abounds in bursæ, and several of them acquire surgical importance from the fact that they frequently become enlarged, and that they are in direct communication with the cavity of the knee joint. The largest and most frequently affected of these is that between the internal condyle of the femur and head of the tibia, and the inner head of the gastrocnemius and semi-membranosus tendon (for shortness called the "semi-membranosus bursa"). This usually, in adults at least, opens into the knee joint. Close by it is a bursa between the semi-tendinosus, and the internal tuberosity of the tibia. This is of less importance surgically. On the outer side the most important bursa is that between the tendon of the popliteus and the external tuberosity of the tibia. It always communicates with the joint. Other three bursæ are fairly constant near this one, but need not be more particularly mentioned. A rheumatic or gouty diathesis predisposes to enlargement of these various bursæ. In most cases a sudden strain, or prolonged muscular exertion is the determining cause. To distinguish such swellings from inflammatory or vascular tumours, their position on the sides of the space, in the vicinity of the above-named tendons, will be observed. Further, an enlarged bursa is firm, elastic, and usually ovoid in outline. It is free from the skin, but slightly movable, and of slow growth. While giving rise to some cramp, stiffness, and weakness in the limb, actual pain is seldom complained of. Fluctuation can be made out when the surrounding structures are relaxed by flexion of the limb, although not detectable in extension. When the knee is partially fixed, some or all of the fluid can frequently be pressed into the joint, and the synovial cavity is felt to distend. It is only when the limb is extended that pulsation of the tumour is detected; it completely disappears on flexion. This is a point of great diagnostic value. There is no bruit or thrill. In a few cases the bursa becomes solid, and in others is the seat of pus formation or of tuberculous disease. Such circumstances materially increase the difficulties of diagnosis. Careful investigation of the history and physical conditions will prevent errors.

**IV. New Growths.**—(*a*) A fatty tumour in the popliteal space will be differentiated from inflammatory affections by its indolent character, from glandular and bursal swellings by its consistence, its irregularly lobulated shape, its mobility in spite of a slight attachment to the skin. Its adventitious pulsation being non-distensile, and the position of the limb, and proximal pressure on the main vessel making no alteration on its size or feeling, will help to distinguish it from aneurism. (*b*) The diagnosis between sarcoma and aneurism is always a difficult one, chiefly from the fact that the sarcoma, in addition to any transmitted pulsation which it may receive from the artery, has an inherent pulsation of its own. Thrill and bruit also belong to both swellings, and although those of the sarcoma are more continuous and murmuring, this is not always sufficient to establish a diagnosis. In sarcoma proximal pressure arrests pulsation and diminishes the size of the swelling, which slowly refills, without the characteristic bound which follows the relaxation in aneurism. Other points which indicate the malignant character of the swelling are its insidious onset, deterioration of the patient's general health out of all proportion to the physical signs, with marked loss of weight. Infiltration of surrounding structures, including the skin, distension of superficial veins, and enlargement of lymphatic glands follow. A new growth seldom has the smooth, regular, and globular outline of an aneurism. When it grows from the interior of the bone, the characteristic egg-shell crackling will aid the diagnosis. (*c*) Having excluded aneurism, it is still necessary to diagnose between sarcoma and gumma of bone. This is done by inquiring into the history of the patient and of his swelling; by observing the effect of the growth on his general condition; and, lastly, by the result of large doses of potassium iodide, and mercury judiciously administered. It is to be borne in mind that even malignant growths sometimes show slight but transient benefit under the action of these drugs.

